# Synergistic Zinc(II) and Formate Doping of Perovskites: Thermal Phase Stabilization of α-FAPbI_3_ and Enhanced Photoluminescence Lifetime of FA_0.8_MA_0.2_PbI_3_ up to 3.7 µs

**DOI:** 10.3390/molecules29020516

**Published:** 2024-01-20

**Authors:** Merk M. Hoeksma, René M. Williams

**Affiliations:** Molecular Photonics Group, van ’t Hoff Institute for Molecular Sciences (HIMS), Universiteit van Amsterdam, Science Park 904, 1098 XH Amsterdam, The Netherlands; meeshoeksma@hotmail.com

**Keywords:** solar cells, formamidinium, lead, iodide, alloys

## Abstract

Adding zinc (II) cations and formate anions improves the thermal phase stability of α-FAPbI_3_ materials, and the spin-coated thin films of such doped FAPbI_3_ (produced using MACl) show an increased emission lifetime of up to 3.7 μs on quartz (for FA_0.8_MA_0.2_PbI_3_). This work investigates the effects of zinc and formate on the phase stability and time-resolved photoluminescence of FAPbI_3_ perovskites for solar cell applications. Perovskite samples with varying concentrations of zinc and formate were made by incorporating different amounts of zinc formate and zinc iodide and were characterized with XRD. Doping levels of 1.7% Zn(II) and 1.0% formate (relative to Pb) seem optimal. The thermal phase stability of the doped perovskite powders (FAPbI_3_) and thin films (FA_0.8_MA_0.2_PbI_3_) was assessed. XRD of the thin films after 6 months shows only the alpha-phase. The time-resolved photoluminescence spectroscopy of the doped spin-coated perovskite samples (FA_0.8_MA_0.2_PbI_3_ produced using MACl) is reported. The results show that synergy between an anionic and a cationic dopant can take place, making the perovskite thermally more phase-stable (not converting to the yellow delta-phase) with a longer charge carrier lifetime. In order to produce good thin films by spin coating, the use of MACl was essential.

## 1. Introduction

Combatting the climate change that started since the industrial revolution is one of today’s most significant challenges [1]. To succeed in this challenge, greenhouse gas emissions must be drastically reduced, and electrification of society and industry is the target. In 2021, there was only a 13% contribution of solar energy to the total energy generated in a renewable way [2]. Improving the cost, efficiency, and availability of solar panels can significantly increase the global amount of electricity obtained from solar energy.

The past two decades have seen a significant increase in research into new materials for solar cells, and one of the most promising candidates is the metal halide perovskite. Since the first report describing the use of these perovskites as light-absorbing material in solar cells in 2009, the power conversion efficiency of the perovskite solar cells has been improved from 3.8% [3] to over 26% in 2023 [4]. Two factors that have significantly contributed to the rise of perovskites in solar cell research are the cheap materials and the relative ease at which devices can be produced. With the focus on performance over the past decade, the challenge now lies in understanding and improving the stability and longevity of perovskite solar cells. A similar operating lifetime as obtained for silicon solar cells should be achieved for market adaptation.

In our previous work, the effect of Zn(II) on the stability [5] and performance [6] of perovskite materials was investigated. It was found that incorporating Zn(II) into the perovskite structure results in improved stability and an increased power conversion efficiency. Zinc is a good dopant for B-site substitution, with 2.5% seeming close to the optimum. It should be noted that interface engineering has been recently reported with Zn(II) materials, giving 23.25% PCE [7].

Regarding X-site doping, formate anions are an excellent option. One of the highest recently published performances was by Jeong et al. [8], who used formate (2%) as an additive and produced a cell with a certified efficiency of 25.2%.

The primary objective of the study presented here is to explore the influence of simultaneous doping with zinc(II) cations and formate anions on the stability and performance of FAPbI_3_-based perovskites. We combine B and X-site doping in formamidinium lead triiodide. Experiments were conducted involving the fabrication of perovskite samples with varying concentrations of zinc and formate. These samples were fabricated by incorporating different amounts of zinc formate and zinc iodide into the precursor solutions and making powdered perovskites (FAPbI_3_) and thin films (FA_0.8_MA_0.2_PbI_3_), the latter characterized with XRD. The performance of the perovskite thin films was evaluated using time-resolved photoluminescence (TR-PL) spectroscopy. TR-PL spectroscopy enables the characterization of the decay dynamics of excited states in materials. By measuring the decay time of the excited states in the perovskite samples, information is gathered about the potential performance of the perovskite in solar cells, focused on radiative charge recombination.

## 2. Results and Discussion

### 2.1. X-ray Diffraction

**Figure 1:**
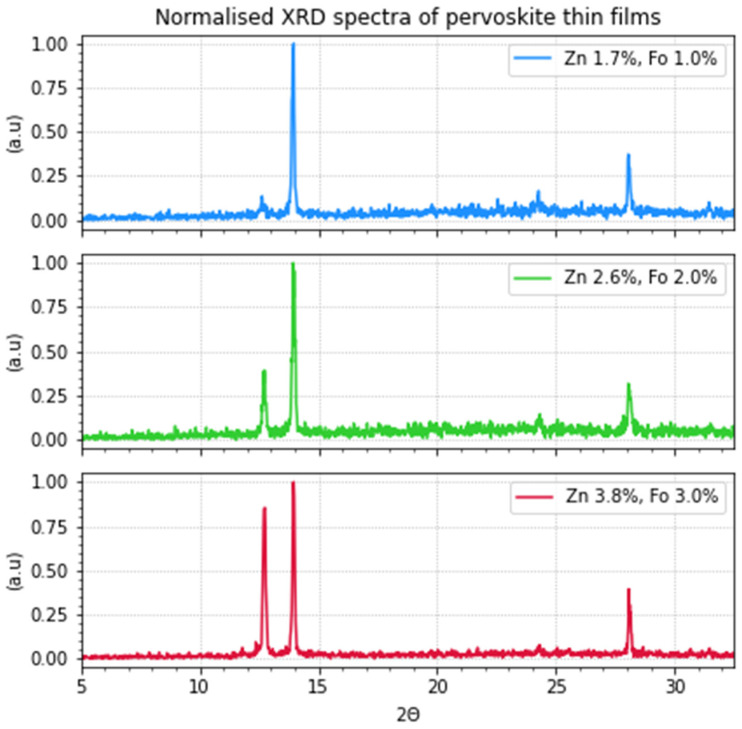
XRD measurements of perovskite thin films (FA_0.8_MA_0.2_PbI_3_) on quartz with varying concentrations of zinc and formate, as indicated. The diffraction peaks of the cubic α-phase at 13.9° and 28.1° are clearly visible. The larger diffraction at 12.7° corresponds to the presence of PbI_2_.

**Figure 2:**
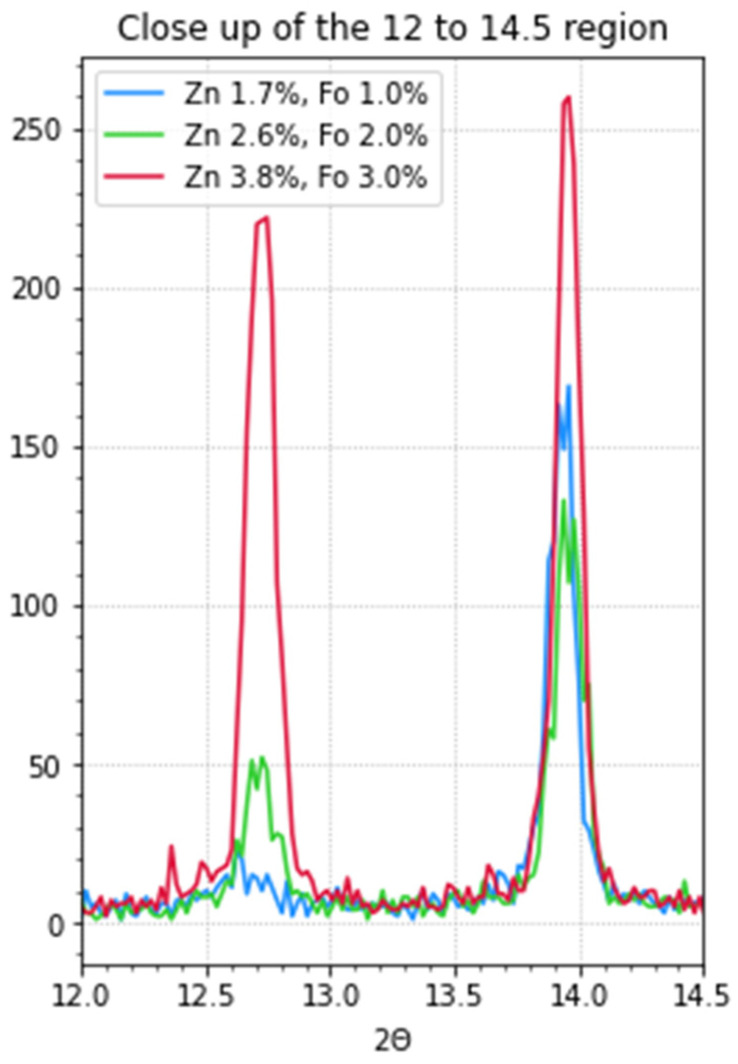
XRD measurements of perovskite thin films on quartz with varying concentrations of zinc and formate. The larger diffraction on the left, at 12.7°, corresponds to the presence of PbI_2_. (Zoomed in relative from Figure 1).

X-ray diffraction (XRD) measurements were performed on thin-layer samples on quartz substrates to obtain a better insight into the composition of the perovskite thin films. These samples were prepared from precursor solutions with zinc formate and zinc iodide. In Figure 1 and Figure 2, the results can be seen. The data are also presented in Table 1. The perovskites display an oriented cubic structure as this is in accordance with the diffractions at 13.9° and 28.1° [5,8,9,10]. The signal at ~24° is also an indication of the perovskite α-phase but indicates some disorder in the crystal packing of the perovskite. The layers that contain more doping have a much higher diffraction at ~12.7° corresponding to PbI_2_ [11,12]. 

### 2.2. Optical Properties of Doped FA_0.8_MA_0.2_PbI_3_ Thin Films

**Figure 3:**
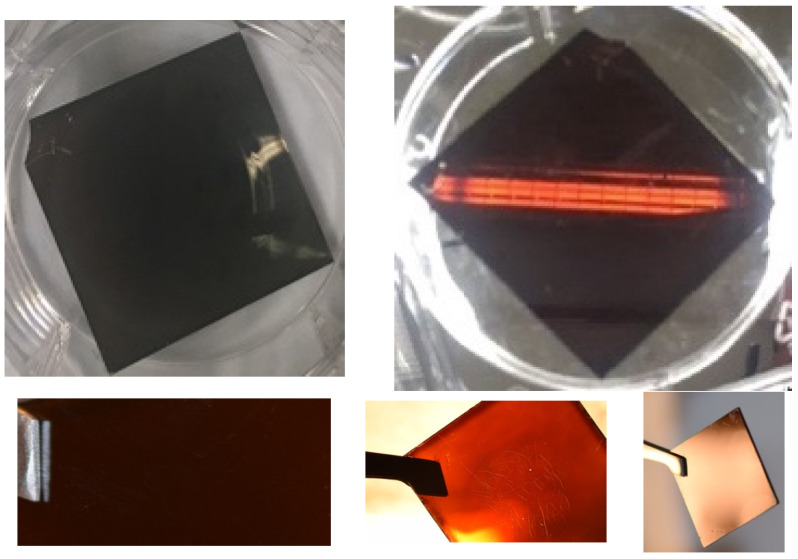
Photographs of representative spin-coated doped perovskite thin films (FA_0.8_MA_0.2_PbI_3_ with 1.7% Zn and 1% Fo) showing their typical appearance: (black) mirror-like in reflective view; transparent brown upon transmissive view at a strong light. The beak of the pincer is 4 mm wide. The substrates are 25 × 25 mm.

A first indication of good film quality can be obtained by visual inspection. Based on our previous work in which films were characterized by electron microscopy, confocal laser scanning microscopy, and optical microscopy, the following can be noted. Figure 3 represents photographs of good films in different lighting conditions using reflective and transmissive observation. The visual appearance of the spin-coated films (FA_0.8_MA_0.2_PbI_3_), as exemplified in Figure 3, changes from a homogeneous dark mirror-like black (as viewed in reflective mode) to a clear conformal shiny brown (when viewed in transmission mode at a bright light, like a fluorescent tube). Such appearance cannot be obtained without using MACl. Inhomogeneous films with swirls, stripes, or large pinholes were discarded.

**Figure 4:**
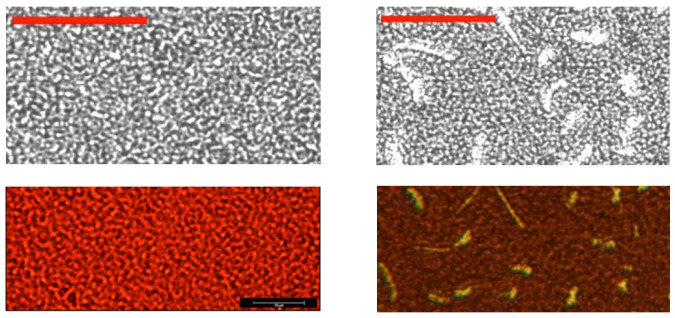
Optical micrographs of two doped films. (**Top left**): Doping with 1.7% Zn and 1% Fo. (**Top right**): Doping with 3.8% Zn and 3% Fo. Scale bar is 28 µm. Note PbI_2_ regions in the right picture (larger white regions). The brown (**lower right**) picture shows the yellow PbI_2_ domains more clearly (same scale as above). The red (**lower left**) micrograph is for comparison, from earlier work [13] on similar films doped with Zn(II) only (with a 10 µm scale bar). This last (red) micrograph is typical for samples of our previous work [5,6,13] that were also studied with SEM and confocal laser scanning microscopy.

Optical microscopy can result in a clear view of multiple-phase domains, containing PbI_2_ (Figure 4) as obtained for high doping (right image). Low doping results in the correct morphology, as compared with earlier studies. The (black and white) optical microscopy images directly correspond to the TR-PL samples (see later). The sample with the lowest doping has a homogeneous phase with ‘worm-like’ features, indicating strands of linked crystals with similar optical properties, as also observed in our earlier work with confocal microscopy (Figure 4 of ref. [6]). This low-doping material shows the longest PL lifetime. The optical microscopy is in agreement with the XRD (showing clear PbI_2_ at higher doping) and shows that a homogeneous phase displays a longer PL lifetime (see later).

**Figure 5:**
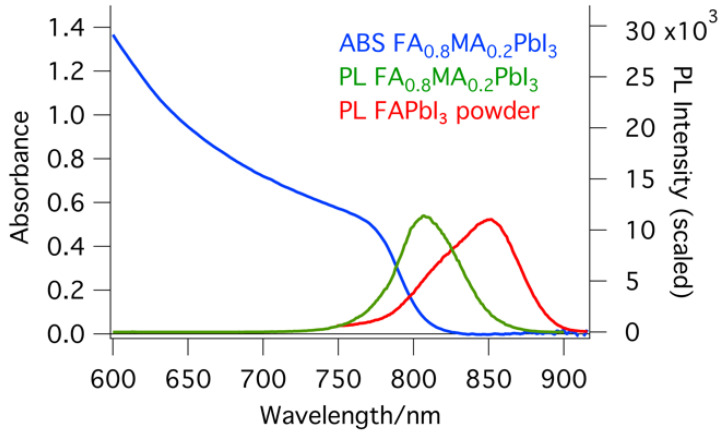
UV–Vis absorption spectrum (blue) as well as photoluminescence spectrum of doped perovskite thin films (FA_0.8_MA_0.2_PbI_3_) with optimal doping (1.7% Zn and 1% Fo, in green) and photoluminescence spectrum of a perovskite powder (FAPbI_3_, with 1.3% Zn and 2.6% Fo, in red).

UV–Vis absorption and steady-state emission spectra (with 810 nm maximum) of good films displayed in Figure 5 show correct features [5,8,13]. Emission spectra of the doped powders show a clear red shift (to 850 nm), in agreement with their constituents (FAPbI_3_). 

Time-resolved photoluminescence (TR-PL) spectroscopic techniques have been widely used to study charge separation and extraction in perovskites [14,15,16]. Two advantages of using TR-PL are as follows: (1) it can be performed without making direct contact to the material; and (2) it allows measurements in which the presence of an HTL and an ETL can be varied [17]. In general, it can be stated that perovskites with longer emission lifetimes on quartz can, in principle, have higher performance [8,14,16].

**Figure 6:**
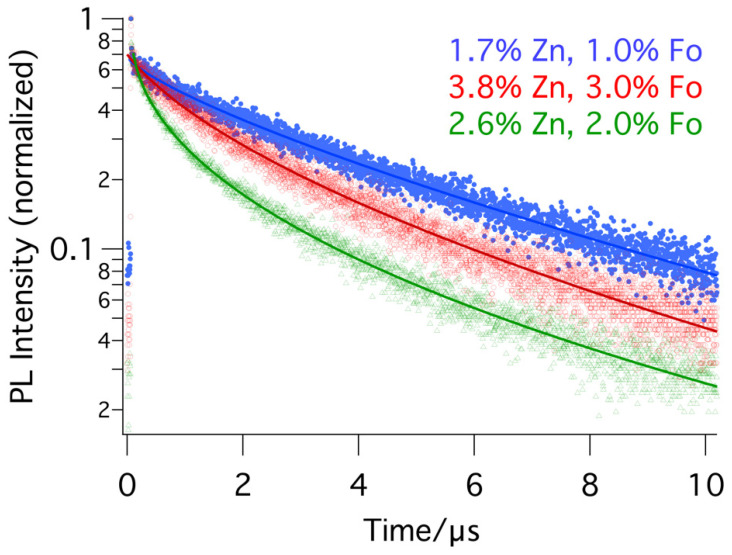
Photoluminescence decays (with stretched exponential fit) of differently doped perovskite thin films (FA_0.8_MA_0.2_PbI_3_). In blue, optimal doping with 1.7% Zn and 1% Fo. In red, non-optimal doping with 3.8% Zn and 3% Fo. In green, non-optimal doping with 2.6% Zn and 2% Fo.

In Figure 6, the results of the TR-PL measurements of three doped samples (FA_0.8_MA_0.2_PbI_3_) are displayed. The details of the fits are combined in Table 2. For each sample, optimal areas of the sample were measured. The dots represent the measurement data, and the lines represent the fits. When comparing the lifetime of the three different samples, 3.7 μs, 0.6 μs, and 1.4 μs for the light, medium, and heavily doped samples, respectively, it can be noted that there is no linear correlation with the doping level. However, clearly, low zinc doping and low formate doping positively affect the emission lifetime of these samples, to levels that have not been reached so far with single component doping (see Table 3).

Comparing lifetimes obtained from literature can be challenging due to the varying models, measuring devices, parameters used, and sample composition, which may include perovskite layers and interface layers. A lifetime of 0.588 μs has been reported [5] for a mixed (FA_0.85_MA_0.15_)Pb(I_2.85_Br_0.15_) film on a quartz substrate using a stretched exponential fit. This is a typical lifetime for undoped FAPbI_3_-based thin-film materials. When Zn(II) was added, this increased to 0.634 μs. Samples in which the crystals were aligned using 3-CPACl had an emission lifetime of 1.547 μs. In the paper by Jeong et al. [8], a specific lifetime is not provided, but in previous work by the same group on Cs_0.05_FA_0.85_MA_0.10_Pb(I_0.97_Br_0.03_)_3_ perovskites with a passivation layer of 4-tert-butyl-benzylammonium iodide (BBAI), an emission lifetime of 2.6 μs was reported [18]. Chen et al. found a lifetime of 16 μs, which is the longest reported [19]. This lifetime was measured for a (FAPbI_3_)_0.9_(MAPbBr_3_)_0.05_(CsPbBr_3_)_0.05_ single crystal perovskite. However, single crystals are not directly comparable with thin films. They are hard to compare because recombination at the perovskite crystal edges usually occurs much faster [17]. The longest lifetime of 3.7 μs reported in this work (FA_0.8_MA_0.2_PbI_3_) is long compared with the thin-film measurements, indicating that a cell with a small amount of zinc and formate doping can, in principle, perform very well. Furthermore, it was found that without the addition of (MABr or) MACl, it was impossible to create good perovskite thin films with spin coating.

To expand on our previous TR-PL overview [6], Table 3 shows photoluminescence lifetimes of perovskite materials from the literature, together with dopant or additive information.

**Table 3:** Overview of long photoluminescence lifetimes reported in the literature for perovskite materials (thin films on quartz, unless indicated differently).

Sample Composition	τ (ns)	Additives/ Specialties	Ref.
Cs_0.05_FA_0.9_MA_0.05_Pb(I_0.9_Br_0.1_)_3_	16,000	Single crystal	[19]
MAPbI_3_	8830	TOPO	[20]
FA_0.8_MA_0.2_PbI_3_	3700	Zn(II), Fo^−^	This work
FA_0.92_MA_0.08_PbI_3_	2835	PEAI, Cl^−^	[6]
Cs_0.05_FA_0.85_MA_0.10_Pb(I_0.97_Br_0.03_)_3_	2600	BBAI	[18]
FA_0.85_MA_0.15_Pb(I_0.95_Br_0.05_)_3_	1547	Zn(II), CPACl	[5]
FA_0.95_MA_0.05_Pb(I_0.95_Br_0.05_)_3_	1105	(I_3_)^−^	[6]
FAPbI_3_	439	HI	[16]
MAPbI_3_	920		[20]

Note that TOPO (tri-octyl phosphine oxide), in our view, is not suitable for large-scale production.

### 2.3. Thermal Stability of Doped Perovskites

**Figure 7:**
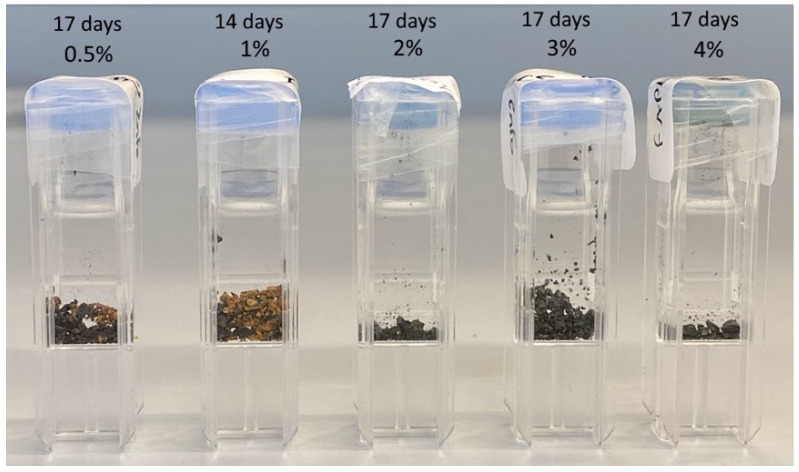

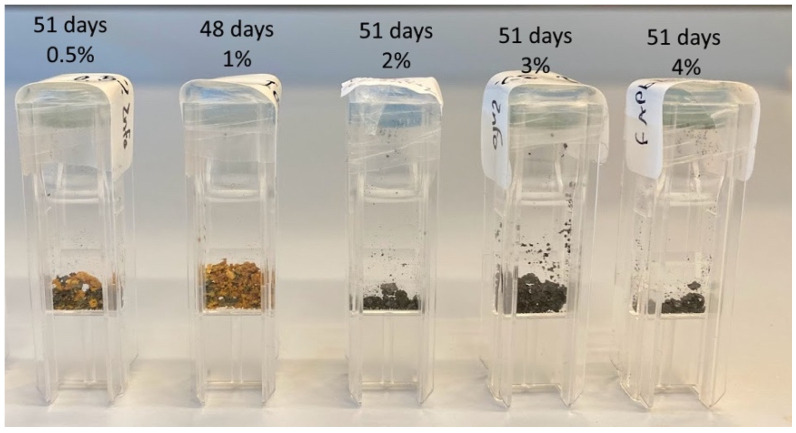
Visual of the discoloration of the black perovskite samples as a function of time. It can be clearly observed that 2% ZnFo_2_, or more, induces thermal stabilization of the black α-phase.

**Figure 8:**
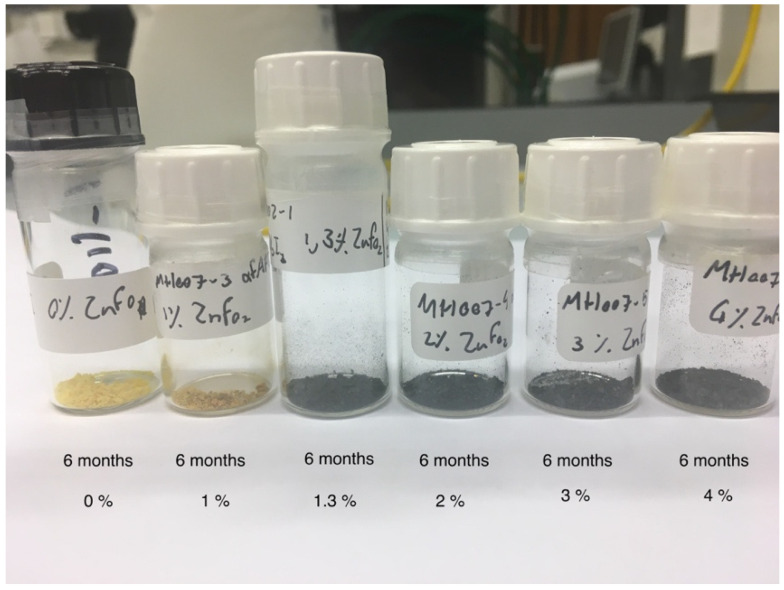
Visual of the discoloration of the black perovskite samples after 6 months. It can be clearly observed that 1.3% ZnFo_2_ doping, or more, induces thermal stabilization of the α-phase. Samples with 0 or 1% doping are yellow after 6 months. Samples with 1.3, 2, 3, and 4% ZnFo_2_ doping remain black. The sample with 1.3% doping was made for extra precision in determining the limit.

**Figure 9:**
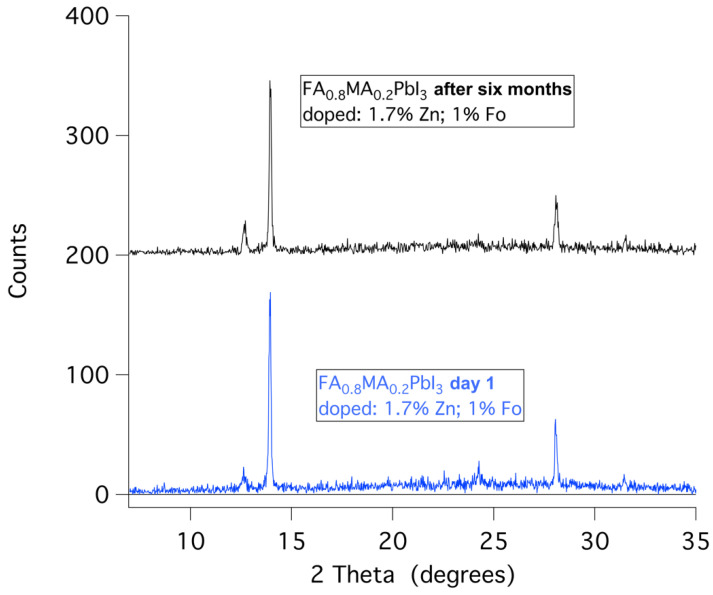
The XRD of spin-coated FA_0.8_MA_0.2_PbI_3_ thin films optimally doped with zinc (1.7%) and formate (1%) direct after preparation (blue) and six months later (black). The XRD pattern proves that the black α-phase is thermally stable.

Considering the fact that the pure black α-phase of FAPbI_3_ perovskite converts to the yellow δ-phase within 24 h, or a few days, a simple straightforward approach to stability is visual color observation. Figure 7 and Figure 8 show examples of typical observations that can be made. Clearly, samples with 0, 0.5, and 1% ZnFo_2_ doping have become yellow (or yellow-brown) over the course of 2 weeks, 7 weeks, or 6 months. However, doping with higher levels of ZnFo_2_ (1.3, 2, 3, and 4%) results in the observation of black powders (Figure 7 and Figure 8) even after half a year. To determine the deterioration of perovskite samples doped with various amounts of zinc formate, first, visual observation was used. The samples were made by drop-casting solutions with varying concentrations of zinc formate added to the FAPbI_3_ precursor solution (see Section 3). The powdered samples are shown in plastic cuvettes (Figure 7) or in regular sample vials (Figure 8). These simple visual observations are corroborated by XRD measurement of the thin film (FA_0.8_MA_0.2_PbI_3_) that shows the longest emission lifetime. Figure 9 displays that the XRD pattern of an aged spin-coated film shows the same results as the visual observations (see also Table 4, for XRD data). The doped perovskite α-phase of FA_0.8_MA_0.2_PbI_3_ is thermally stable after six months (black trace) of storage under ambient conditions in a desiccator with regular indoor artificial light (humidity free, no sunlight).

## 3. Experimental

### 3.1. FAPbI_3_ Synthesis

The synthesis of FAPbI_3_ was performed using the method published by Tong et al. [21]. Formamidinium acetate (1.05 g) was dissolved in 1.5 mL of HI in H_2_O solution (57 wt.%). While stirring, PbI_2_ (4.61 g) was added to the solution. Not everything dissolved, and then 5 mL of GBL (gamma butyrolactone) was added. When everything had dissolved, the solution was heated at 95 °C for 1 h. The yellow-orange crystals were filtered using a frit filter and reduced pressure and then transferred to a round-bottom flask and heated at 150 °C for 2 h. The black spikey crystals were then thoroughly washed using diethyl ether, and the diethyl ether was removed using vacuum filtration. The washing was performed by transferring the crystals to the frit filter and then submerging them in diethyl ether. Using a spatula, the larger crystals were broken up. The crystals were transferred to another round-bottom flask and heated for another three hours at 150 °C. Black crystals were collected. In general, these black α-FAPbI_3_ crystals will turn yellow within 24 h, even in the dark. If not properly washed with diethyl ether, traces of acetate can remain in the FAPbI_3_ product.

It can be noted that lead acetate is used in methyl ammonium-based perovskites as an additive to slow down the crystallization in thin films, for example, leading to larger grains and fewer pinholes in the crystal structure [22]. More recently, lead acetate was used as a starting material for making perovskite layers with a mix of formamidinium and cesium. In this example, the crystallization is also controlled by the acetate by evaporating the acetate as ammonium acetate (the ammonium is added as a salt with the X anion) [23].

### 3.2. Precursor Solution Preparation for Spin Coating

A general procedure for the preparation of precursor solutions for spin coating is as follows. An amount of FAPbI_3_ was added to a sample flask. According to this amount, the amount of ZnFo_2_ and ZnI_2_ that should be added for the desired doping percentages is calculated and subsequently added, as well as other additives. The additives are calculated in molar percentages compared with the amount of lead in the base FAPbI_3_. Subsequently, solvent is added, and the solution is mixed with a small magnetic stirring rod until dissolved. To make sure all solids have dissolved, the solution is also sonicated.

The typical compositions (see Table 5) and procedures for the precursor solutions used in the experiments are given below. Note that MACl is added to aid the formation of thin films (FA_0.8_MA_0.2_PbI_3_). Without MACl, we were unable to produce films of good quality. In the literature, MACl is also often added in quantities up to 35 mol% [8]. An alternative that was also tested is MABr, but this induces Br incorporation.

### 3.3. Perovskite Powders

The precursor solutions for the preparation of the perovskite powders were made by dissolving the amounts of ZnFo_2_ and FAPbI_3_ that can be seen in Table 6 in 0.15 mL of DMSO. To fully dissolve the solids, the mixture was sonicated for up to 40 min at 35 °C. The goal was to obtain the highest concentration possible.

### 3.4. Thin Films for XRD and TR-PL (FA_0.8_MA_0.2_PbI_3_)

Three different precursor solutions were made for the spin coating, with a low, medium, and high amount of doping. The different precursor solutions were made by dissolving the specified amounts of FAPbI_3_, ZnFo_2_, ZnI_2_, and MACl in 1.1 mL of a 1:4 mixture of DMSO:DMF.

The solutions were stirred using a small magnetic stirring rod for 25 min and also sonicated for 25 min until everything had dissolved. To make sure no particulates would be present in the spin-coating process, the solutions were filtered using PTFE-syringe filters.

Similar to what has been reported in the literature, precursor solutions that have aged (e.g., for 30 days) produce layers of lower quality [24].

### 3.5. Doped Powder Preparation (FAPbI_3_)

The preparation of the precursor solutions for the doped perovskite powders can be found in the previous section. The solutions were drop-cast into Petri dishes in tiny drops using a syringe and a needle. The Petri dishes were heated at 180 °C directly on a hotplate. The small drops turned black, and some vapor escaped; using a spatula, the drops were then scraped off and made into a powder. The minimum time was 20–25 min for the drops to dry enough to scrape them off. Using smaller drops helps in drying properly.

### 3.6. Substrate Cleaning

All substrates were cleaned by sonicating in a beaker with milli-Q water with Helmanex III soap for 15 min. The substrates were then rinsed with milli-Q water and placed back in a rinsed Teflon sample holder. After this, the samples were sonicated for 15 min in acetone and subsequently in 2-propanol also for 15 min. After sonication, the substrates were dried with compressed air and lastly treated with ozone in a UV-ozone oven for at least a half hour.

For all of the cleaning steps, it is best if the slides are placed in the sample holder in a way that there is the least amount of contact possible between the sample holder and the substrates. This is carried out because, sometimes, especially when using thicker substrates, the substrates sit tight in the slots and will not get properly cleaned. This can later show up during spin coating, leading to uncoated edges on the substrates. To ensure that the sample holders do not affect the cleaning in the UV-ozone oven, the substrates can be laid on a clean glass plate in the UV-ozone oven with the side where the perovskite will be deposited facing up. Quartz substrates were obtained from Osilla. Glass plates from Menzel were also used.

### 3.7. Spin Coating

The thin-film samples were produced by spin coating. The techniques and parameters used during spin coating were varied and experimented with. A very useful resource used was the Osilla spin-coating guide [25]. The procedure that was ultimately found to reliably produce good quality films is as follows. For spin coating, the substrates are taken out of the UV-ozone oven one by one, and the oven is switched on again for the remaining substrates. The substrate is centered on the table by eye, and the suction is switched on. Then, on the spin coater, a gentle stream of N_2_ is blown over the substrate for about three seconds to remove any possible dust particles. At 3000 rpm, with no ramp, the sample was spun for 30 s; then, after 3–4 s of spinning, 0.06 mL of precursor solution was put on the center of the spinning substrate as close as possible but without touching the surface; this can be performed using a micropipette or a syringe and needle, but when using a needle, extra care has to be taken that the solution is not applied too fast. The micropipette was found to create less dripping. After spinning for 30 s, the sample is spun for another 30 s at 2500 rpm. After a few seconds, 0.7–0.8 mL of diethyl ether (DEE) is applied to the surface using a syringe and a needle. The syringe’s needle is held as close to the surface as possible and in the center of the spinning sample. The plunger is pushed down in a couple of seconds, with the goal of the DEE stream not being so hard that it ‘blasts’ the perovskite layer away but also not so slow that it drips. After this anti-solvent step, the sample is taken from the spin coater and put onto a hotplate to anneal for 20 min at 150 °C.

If a little MABr (or MACL) is added, this makes a large difference in the crystallization properties of the thin films. Without it, the films would not turn dark-brown/black when put on the hotplate for annealing but stay yellow and become a bit darker yellow over time. With MABr or MACl, the thin films would turn from light yellow to brown within a second. It was found that you need more MACl than MABr to obtain a similar effect in crystallization.

### 3.8. Time-Resolved Photoluminescence

Time-correlated single-photon counting measurements of the perovskite thin films on quartz were carried out on an in-house built setup, PicoQuant PDL 828 “Sepia II” and a PicoQuant HydraHarp 400 multichannel picosecond event timer and TCSPC module. A laser (PicoQuant LDH-D-C-485, PicoQuant, Berlin, Germany) with a wavelength of 485 nm was used for excitation with a repetition rate of 0.1 MHz. The emission was measured with a single-photon avalanche diode (SPAD) detector (Micro Photon Devices, MPD-5CTD, Micro Photon Devices S.R.L., Bolzano, Italy), and to remove the laser light, a long-pass filter (Thorlabs FEL-488, Thorlabs Inc., Newton, NJ, USA) was used. The laser power at 100% was around 15 mW. Also, an ND4 filter was used to decrease the intensity. This setup was used as it was possible to record up to 10 μs, which was necessary for the long lifetimes on quartz substrates.

The photoluminescence (PL) lifetime of perovskite layers is determined by fitting the TR-PL data to a mono-exponential, bi-exponential, or stretched-exponential model [17]. The model used to fit the data depends on the measured sample, energy levels, and electron transfer process in a ‘standard’ perovskite solar cell [26].

A bi-exponential model is often used when measuring samples containing an HTL or an ETL [27]. In case no charge extraction layers are present on the sample, a stretched exponential was chosen to fit the data to the function $f\left(t\right)=y_{0}+A{\;e}^{{\left(-t/\tau \right)}^{\beta }}$, where *A* is the amplitude, *τ* is the lifetime, and *β* is the stretch factor (0 < *β* < 1). The stretch factor signifies the heterogeneity of the system. If *β* is equal to 1, the function converts to a mono-exponential function. All photophysical data reported here have an estimated error of 5 to 10%.

### 3.9. XRD Measurements of Thin Films

XRD measurements were performed on thin films on quartz. These were the same samples that were used in the time-resolved photoluminescence measurements. The measurements were performed using a Bruker D2 phase diffractometer operated at 30 kV and 10 mA using a Cu source. The substrates were scanned between 2θ = 5° and 2θ = 60° with a step size of 0.202°. Data analysis was performed using Python (version 3.10.8) and Excel (version 16.16.27).

### 3.10. Software Used

For the analysis of the data, fitting, and plotting of the data, Python programming language was used (version 3.10.8) [28]. For fitting the TCSPC data, the curve fit function from the SciPy library was used. The raw and modified data and the Python code used to make the figures can be found in the accompanying Supporting Materials.

## 4. Conclusions and Future Outlook

Simultaneous zinc formate and zinc iodide doping of perovskite precursor solutions influences the thermal phase stability and the PL lifetime of FAPbI_3_-based perovskites in a positive way.

TR-PL measurements of FA_0.8_MA_0.2_PbI_3_ materials show that the samples with low doping levels (with 1.7% zinc and 1% formate) have the longest lifetimes, 3.7 μs, using a stretched exponential fit.

The effect of zinc and formate on the phase stability of perovskite powders and thin films was investigated. For the powders (FAPbI_3_), it was found that between 1.3% and 4% ZnFo_2_ doping is where the phase stability is affected: the black α-phase does not convert to the yellow δ-phase. In the thin films (FA_0.8_MA_0.2_PbI_3_) with low doping (1.7% Zn and 1% Fo), no phase change was observed: XRD after 6 months shows a stable α-phase.

Increasing the dopant concentration led to the presence of PbI_2_ in the films. To be able to tune the ratio of zinc and formate added to the precursor solution, Zn(CHOO)_2_ and ZnI_2_ are added. Zinc iodide is added to be able to make ratios other than 1:2 = zinc:formate. In the XRD measurements (Figure 1 and Figure 2), the increase in PbI_2_ is identifiable with an increase in the dopant concentration in the precursor solution. The observation that the presence of PbI_2_ is detrimental to the performance is not new, as it is known to be one of the degradation products [12].

Overall, it was found that zinc and formate positively affect the thermal phase stability of the perovskite α-phase and PL lifetime of FAPbI_3_-based materials. In the grand scheme of events, we note that optimal individual doping contents for perovskites (e.g., 2.5% Zn(II) and 2% Fo) do not correspond to optimal contents for synergistic doping agents (1.7% Zn and 1% Fo). Our work indicates that the sum of added doping components should be ~3% (or less). Fine-tuning the ratio of Zn(II) to Fo within this 0.1 to 3% total doping window may lead to further improvements of the material properties of perovskites for single junction or tandem cells [29,30].

## Figures and Tables

**Table 1:** The results corresponding to the XRD measurements of the three doped samples (FA_0.8_MA_0.2_PbI_3_). The corresponding graphs can be seen in Figure 1 and Figure 2. The diffraction angles are indicated, the percentage is the normalized height of the spectra, and all spectra are normalized relative to the highest peak. The peak amplitude is the value as measured (not normalized). The FWHM is the full width at half maximum of the peaks; for the very small peaks, it was not possible to calculate this.

Sample Code	Peak (°)	%	Peak Amplitude	FWHM (°)	Designation
**1**	12.7	13.6	23	-	PbI_2_
	13.9	100	169	0.1263	α-phase
	24.3	16.6	28	-	α-phase
	28.1	37	63	0.1273	α-phase
**2**	12.7	39	52	0.1415	PbI_2_
	13.94	100	133	0.1425	α-phase
	24.3	14	19	-	α-phase
	28.1	31.5	42	0.1727	α-phase
**3**	12.36	7.7	20	-	PbI_2_
	12.7	85	221	0.153	PbI_2_
	13.96	100	260	0.118	α-phase
	~24	7.7	20	-	α-phase
	28.1	39	102	0.0809	α-phase

**Table 2:** The details of doping and the results of the fitting of the TR-PL data with a stretched exponential (FA_0.8_MA_0.2_PbI_3_).

Sample Code	% Zn	% Fo	% Total	τ (ns)	β
**1**	1.7	1.0	2.7	3700	0.77
**2**	2.6	2.0	4.6	610	0.46
**3**	3.8	3.0	6.8	1420	0.58

**Table 4:** The results corresponding to the XRD measurements of the sample of Figure 8, (FA_0.8_MA_0.2_PbI_3_) doped with zinc (1.7%) and formate (1%). The diffraction angles are indicated, the percentage is the normalized height of the spectra, and all spectra are normalized relative to the highest peak. The peak amplitude is the value as measured (not normalized). The FWHM is the full width at half maximum of the peaks; for the very small peaks, it was not possible to calculate.

Sample Code	Peak (°)	%	Peak Amplitude	FWHM (°)	Designation
**1**	12.7	13.6	23	-	PbI_2_
	13.9	100	169	0.1263	α-phase
	24.3	16.6	28	-	α-phase
	28.1	37	63	0.1273	α-phase
**1 (aged)**	12.7	20	29	0.1783	PbI_2_
	13.9	100	146	0.1356	α-phase
	24.3	12	18	-	α-phase
	28.1	34	50	0.1941	α-phase

**Table 5:** The contents of the precursor solutions for the preparation of the spin-coated perovskite films (FA_0.8_MA_0.2_PbI_3_) with increasing concentrations of zinc formate. On the left side of the table are the amounts that were added to the precursor solution. On the right side of the table, the calculated mol% compared with the amount of Pb is displayed as well as the sum of Zn and Fo doping.

Sample Code	FAPbI_3_ (mg)	ZnFo_2_ (mg)	ZnI_2_ (mg)	MACl mg (mol%)	% Zn	% Fo	% Total
**1**	800.2	0.9	5.2	18.5 (21.7)	1.7	1.0	2.7
**2**	806.5	2.0	6.9	21.2 (24.6)	2.6	2.0	4.6
**3**	806.1	3.0	9.3	19.9 (23.1)	3.8	3.0	6.8

**Table 6:** The contents of the precursor solutions for the preparation of the FAPbI_3_ perovskite powders with increasing concentrations of zinc formate.

Sample Code	FAPbI_3_ (mg)	ZnFo_2_ (mg)	% Zn	% Fo	% Total
0.0%	295.6	0.0	0.00	0.00	0.00
0.5%	299.0	0.3	0.41	0.82	1.23
1.0%	297.8	0.7	0.96	1.92	2.88
2.0%	297.3	1.7	2.33	4.66	6.99
3.0%	297.0	2.2	3.02	6.04	9.06
4.0%	298.2	2.9	3.96	7.92	11.88

## Data Availability

Data are contained within the article and Supplementary Materials.

## Supplementary Materials

Data files, analysis files, and all files related to this research (RDM) are available here: https://doi.org/10.21942/uva.24757407.v1 (accessed on 19 January 2024).

## Abbreviations

CPACl	3-chloropropylamine hydrochloride
DEE	diethyl ether
PL	photoluminescence
PTFE	polytetrafluoroethylene
DMF	dimethylformamide
DMSO	dimethylsulfoxide
GBL	gamma butyrolactone
τ	lifetime of an excited state
MABr	methylammonium bromide
MACl	methylammonium chloride
TR-PL	time-resolved photoluminescence
Fo	formate anion (HCO_2_)^-^
FAPbI_3_	formamidinium lead tri-iodide
TOPO	tri-n-octylphosphine oxide
XRD	X-ray diffraction
